# Dynamics of Peroxisome Homeostasis and Its Role in Stress Response and Signaling in Plants

**DOI:** 10.3389/fpls.2019.00705

**Published:** 2019-06-04

**Authors:** Tong Su, Wenjing Li, Pingping Wang, Changle Ma

**Affiliations:** Shandong Provincial Key Laboratory of Plant Stress, College of Life Sciences, Shandong Normal University, Jinan, China

**Keywords:** *Arabidopsis*, pexophagy, peroxisome biogenesis, retrograde signaling, stress

## Abstract

Peroxisomes play vital roles in plant growth, development, and environmental stress response. During plant development and in response to environmental stresses, the number and morphology of peroxisomes are dynamically regulated to maintain peroxisome homeostasis in cells. To execute their various functions in the cell, peroxisomes associate and communicate with other organelles. Under stress conditions, reactive oxygen species (ROS) produced in peroxisomes and other organelles activate signal transduction pathways, in a process known as retrograde signaling, to synergistically regulate defense systems. In this review, we focus on the recent advances in the plant peroxisome field to provide an overview of peroxisome biogenesis, degradation, crosstalk with other organelles, and their role in response to environmental stresses.

## Introduction

Peroxisomes, which are found in all eukaryotic cells, are highly metabolic organelles surrounded by a single membrane (De Duve and Baudhuin, 1966; Hu et al., 2012). In plant cells, peroxisomes play vital roles in metabolism since they house many processes including fatty acid β-oxidation, glyoxylate cycles in seedlings, photorespiration in leaves, urate sulfite and polyamine metabolism (Hu et al., 2012), and biosynthesis of phytohormones such as indole-3-acetic acid, jasmonic acid, and salicylic acid (Reumann et al., 2004; Koo et al., 2006; Strader and Bartel, 2011; Kao et al., 2018). In addition, antioxidant enzymes and enzymes which generate reactive oxygen species (ROS) or reactive nitrogen species (RNS) are essential components in peroxisomes for maintaining redox homeostasis (Corpas et al., 2017). Metabolite exchange between peroxisomes and other organelles is important for peroxisomal function and for morphological changes which occur under specific physiological and environmental conditions (Linka and Theodoulou, 2013).

**Figure 1:**
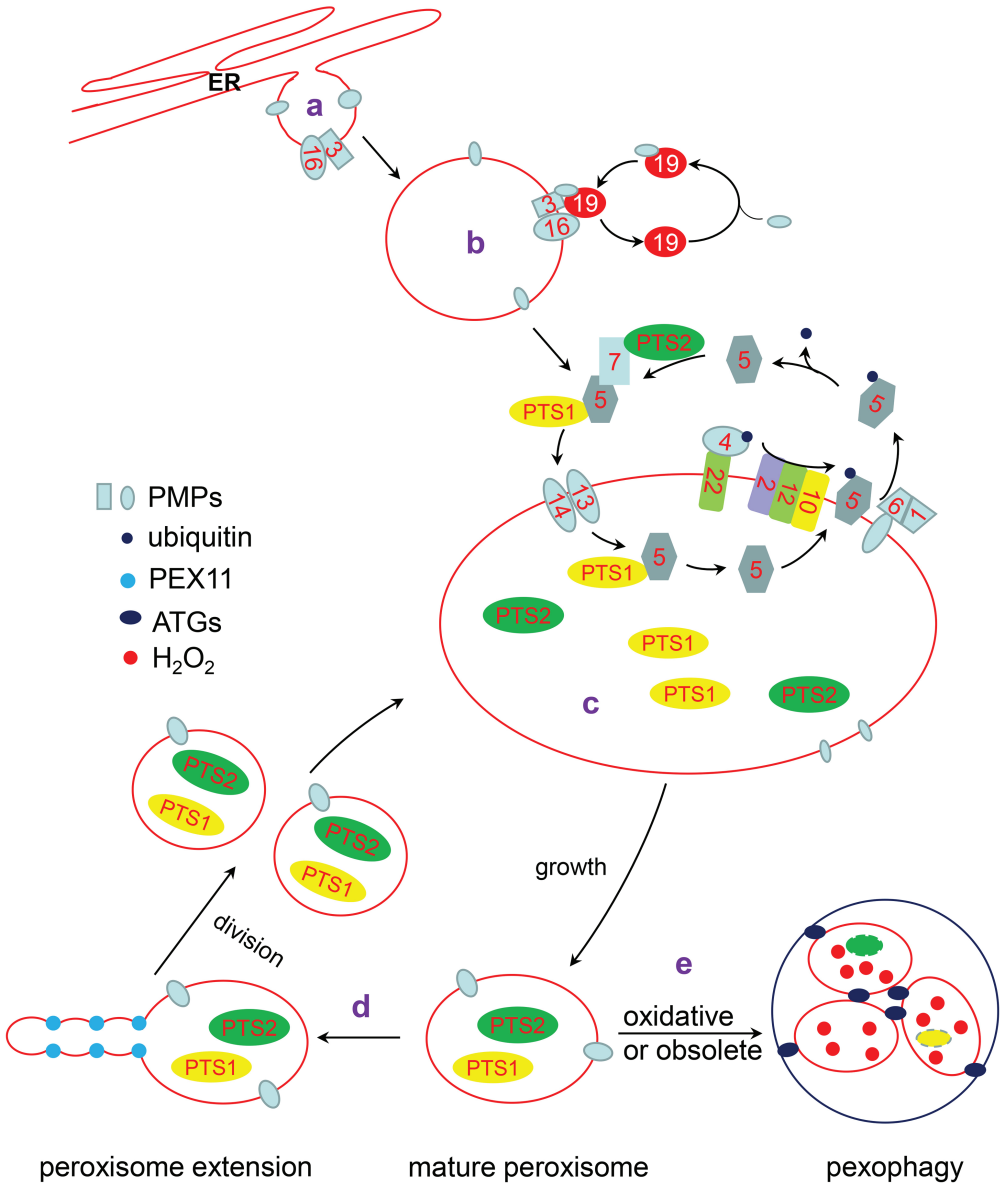
The life cycle of peroxisome. a: Some PMPs are targeted to the ER and localized in special ER subdomains, where new preperoxisomes subsequently bud off. b: The remainder of PMPs are directly inserted into the peroxisomal membrane through a PEX3/PEX19-dependent pathway or another unknown pathway. c: PTS1 and PTS2 proteins interact with their receptor, PEX5 or PEX7, respectively. The receptor/cargo complex is then transported into the peroxisome lumen through the docking complex, composed of PEX13 and PEX14 in *Arabidopsis*. After unloading its cargo protein, PEX5 is mono-ubiquitinated by the PEX4/PEX22 (E2 ubiquitin conjugases) and PEX2/PEX12/PEX10 (E3 ligases) complexes. Subsequently, Pex5 is retrotranslocated to the cytosol by the PEX1/PEX6 complex for recycling. d: Mature peroxisomes elongate and divide into two new peroxisomes. e: Oxidative or obsoleted peroxisomes can be degraded by pexophagy under the action of ATGs.

Peroxisomes are usually oxidized and damaged by metabolic byproducts or environmental stresses. Excess and/or damaged peroxisomes are removed by autophagy to ensure the normal operation of the cell. According to the environmental conditions, the number and quality of peroxisomes in cells can change rapidly and is precisely regulated to maintain peroxisomes homeostasis (Farmer et al., 2013; Yoshimoto et al., 2014; Young and Bartel, 2016). Herein, we review recent advancements in our understanding of peroxisome biogenesis including the mechanisms of import of peroxisomal membrane proteins (PMPs) and peroxisomal matrix proteins, the connection of peroxisomes with other organelles, and the dynamics of how redox status in plants regulates peroxisome homeostasis and peroxisomal-derived retrograde signaling (Figure 1).

## Peroxisome Biogenesis

### Origin of Peroxisomes

It has been reported that peroxin proteins encoded by *PEX* genes are required for peroxisome biogenesis and conserved in yeast, mammals, and plants (Ma et al., 2011; Hu et al., 2012). However, how peroxisomes form has been debated for over three decades. Early models of peroxisome biogenesis suggested that peroxisomes are autonomous organelles that divide by growth and division of pre-existing peroxisomes (Lazarow and Fujiki, 1985; Agrawal and Subramani, 2016). According to this model, a pre-existing peroxisome grows and divides asymmetrically into two new peroxisomes when a certain size threshold is reached (Akşit and van der Klei, 2018). This view was supported by the discovery of peroxisome protein import machinery and the ability of peroxisomes to divide (Lazarow and Fujiki, 1985; Kim and Hettema, 2015). Studies in plants also support the growth and division model (Hu et al., 2012). In *Arabidopsis*, proteins participating in peroxisomal protein targeting and peroxisome division have been identified and characterized (Woodward and Bartel, 2005; Zhang and Hu, 2008; Aung and Hu, 2011; Woodward et al., 2014).

However, the growth and division model does not account for the source of membrane lipids required for the growth of dividing peroxisomes. In *Saccharomyces cerevisiae*, it was shown that lipids are directly transferred from the ER to peroxisomes by a vesicle-independent pathway (Raychaudhuri and Prinz, 2008). In addition, in *S. cerevisiae pex3* and *pex19* cells, which are devoid of detectable peroxisomes, new peroxisomes could form when a wild-type copy of PEX3 or PEX19 was reintroduced into the corresponding mutants, signifying *de novo* peroxisome formation (Hohfeld et al., 1991). Research on *de novo* peroxisome formation in plants is scarce due to the complicacy of plant systems. However, we have gained insights from experiments in yeast. It was shown that *S. cerevisiae* Pex3 is delivered to the peroxisomes through the ER (Hoepfner et al., 2005). Pex3, along with Pex19, is involved in the direct insertion of some PMPs into the ER and the pre-peroxisomal vesicle membrane (Hu et al., 2012; van der Zand et al., 2012). Subsequently, theses pre-peroxisomal vesicles fuse to form a new peroxisome where these PMPs are naturally transferred into the peroxisomal membrane (van der Zand et al., 2010, 2012).

It now seems that neither *de novo* synthesis from the ER nor the growth and division model can fully explain all aspects of peroxisome biogenesis (Smith and Aitchison, 2013; Hettema et al., 2014). Although it is still heavily debated (Knoops et al., 2014; Wroblewska et al., 2017), it has been proposed that the generation of peroxisomes includes *de novo* biogenesis, during which pre-peroxisomal vesicles fuse to form a new peroxisome or fuse with preexisting peroxisomes. This is followed by growth to form mature peroxisomes, which can divide into new peroxisomes (Hu et al., 2012).

### The Transport of Peroxisomal Membrane Proteins

Although the mechanism is not fully understood, there are two possible pathways by which PMPs are imported into peroxisomes (Theodoulou et al., 2013; Erdmann, 2016; Yuan et al., 2016): (1) PMPs are synthesized in the cytosol and imported directly into existing peroxisomes or (2) PMPs are integrated into the ER and subsequently targeted to peroxisomes.

PMPs directly imported into peroxisomes from cytosol need to be recognized by Pex19, a cytosolic PMP receptor that interacts with and stabilizes PMPs (Hettema et al., 2000; Rottensteiner et al., 2004). Cargo-bound Pex19 interacts with peroxisomal membrane-associated Pex3 for the subsequent docking of PMPs. Both Pex3 and Pex19 are necessary for PMP import and peroxisome biogenesis. There are two isoforms of both PEX3 and PEX19 in *Arabidopsis*, which are homologs of yeast Pex3 and Pex19, respectively (Hunt and Trelease, 2004). *Arabidopsis* deficient in PEX19 have enlarged peroxisomes (Nito et al., 2007). In mammals, PEX16 may act as a membrane receptor and, together with PEX19, mediates the direct insertion of PEX3 into peroxisome membranes (Matsuzaki and Fujiki, 2008). The transport of PEX3 to peroxisomes is reduced in mammalian cells with PEX16 knocked down (Matsuzaki and Fujiki, 2008; Aranovich et al., 2014; Hua and Kim, 2016). The null mutation of PEX16 results in non-detectable peroxisome remnants in mammalian cells (Honsho et al., 1998). In *Arabidopsis*, *Shrunken SEed 1* (*SSE1*) mutants deficient in PEX16 were found to show a shrunken seeds phenotype as a result of the disordered formation of oil bodies during seed development (Lin et al., 1999, 2004). *Arabidopsis pex16i* mutants have enlarged peroxisomes, which are reduced in number and show functional defects (Nito et al., 2007). AtPEX16 is located on the membrane of both peroxisomes and the ER and may act as an integral membrane-bound receptor for PEX3 and other PMPs (Karnik and Trelease, 2005; Kim and Mullen, 2013). There are other PMPs, such as PMP22, which are directly inserted into the peroxisome membrane after being synthesized in the cytosol in *Arabidopsis* (Murphy et al., 2003). *Arabidopsis* PMP22 is a homolog of mouse PxMP2, which is a channel protein for small metabolites (Rokka et al., 2009). In addition, *Arabidopsis* PEX2 and PEX10 have been shown to insert into peroxisome membranes directly from the cytosol (Sparkes et al., 2005).

There is increasing evidence that some PMPs are transported to peroxisomes through the ER membrane. An important subgroup of these PMPS is known as the tail anchored (TA) proteins, which are anchored to the peroxisome membrane at the C-terminus and have a short luminal domain with the remaining N-terminus in the cytosol (Cross et al., 2016). In *S. cerevisiae*, Pex15, a TA protein containing a single transmembrane domain near its C-terminus, is targeted to the ER membrane by the guided entry of tail-anchored (Get) pathway (Okreglak and Walter, 2014). The C-terminal of yeast Pex15 contains a PEX19-binding site, which positions Pex15 on the ER membrane through the Get pathway, indicating that Pex15 is inserted into the peroxisome membrane *via* the ER (Halbach et al., 2006; Schuldiner et al., 2008). In plants, the initial evidence of ER-to-peroxisome trafficking emerged from *in vitro* experiments using cottonseed ascorbate peroxidase (APX), a TA protein targeted to the peroxisome membrane (Mullen et al., 1999). It was shown that the post-translated cottonseed APX inserts into ER microsomal membranes, but not other organelle membranes, while over-expressed cottonseed APX was located in both peroxisomes and the ER membrane (Mullen et al., 1999). *Arabidopsis* APX3 has also been detected in ER subdomains, indicating that APX3 is imported to the peroxisome *via* the ER-to-peroxisome pathway (Lisenbee et al., 2003).

### Matrix Protein Entry Into the Peroxisome Lumen

Peroxisomal matrix proteins are synthesized in the cytosol and imported into the matrix of peroxisomes. The peroxisomal matrix proteins are distinguished by their peroxisomal targeting signals (PTSs), which are recognition sequences at the C-terminus (PTS1) and N-terminus (PTS2) of the respective cargo proteins. Typical PTS1 sequences consist of a highly conserved tripeptide [S/A/C]-[K/H/R]-L (Elgersma et al., 1996; Reumann et al., 2004, 2016). The consensus for a PTS2 sequence is R/K-L/V/I-X5-H/Q-L/A (Petriv et al., 2004).

In *Arabidopsis*, two peroxins, PEX5 and PEX7, are responsible for the recognition of PTS1 and PTS2 proteins, respectively. The disordered N-terminal region of PEX5 interacts with PEX7 and components of the docking subcomplex, such as PEX14, while the conserved C-terminal region contains a series of tetratricopeptide repeats (TPR) that bind to the PTS1 of cargo proteins (Zolman et al., 2000; Nito et al., 2002). The WD domain of PEX7 is responsible for its recognition of PTS2 proteins (Woodward and Bartel, 2005). Conserved across eukaryotes, the PTS1 import pathway is the predominant mechanism for cargo proteins to enter plant peroxisomes. However, unlike in yeast, the import of PTS1 and PTS2 proteins are interconnected in plants. In *Arabidopsis,* the binding of PEX7 to PTS2 proteins requires PEX5 as a co-receptor (Woodward and Bartel, 2005), which is necessary for the proper interaction of PEX7 with PTS2-cargo proteins. Mutation of PEX5 (S318L) causes PEX5 to inefficiently bind with PEX7 and disrupts the import of PTS2 proteins, indicating that the PEX5-PEX7 interaction is necessary for PTS2 protein import (Woodward and Bartel, 2005). Additionally, the import of PTS2 proteins is restored by expressing the N-terminus of PEX5 in the PEX5 (S318L) mutant, indicating that the N-terminal domain of PEX5 is necessary for PTS2 import (Khan and Zolman, 2010). Interestingly, a mutation in PEX7 (T124I) results in the reduction of the protein levels of both PEX7 and PEX5 as well as reducing import of PTS1 and PTS2 cargoes, further suggesting that the PTS1 and PTS2 targeting pathways are interconnected in plants (Ramon and Bartel, 2010).

The delivery of peroxisomal matrix proteins requires the action of a large peroxisomal membrane complex known as the importomer, which is composed of the docking subcomplex (Pex13, Pex14, and Pex17) and the RING (Really Interesting New Gene) subcomplex (Pex2, Pex10, and Pex12) bridged by Pex8 in *S. cerevisiae* and Pex3 in *P. pastoris* (Agne et al., 2003; Rayapuram and Subramani, 2006). However, it is not well established how the importomer is organized in plants. The cargo-receptor complex associates with the peroxisomal membrane through the docking subcomplex of the peroxisomal importomer. In *Arabidopsis*, PEX5 can interact with PEX14 but not with PEX13, and the WxxxF/Y (Trp-X-X-X-Phe/Tyr) pentapeptide repeats in PEX5 are crucial for this interaction (Nito et al., 2002). PEX13, but not PEX14, is responsible for the interaction with PEX7 in *Arabidopsis* (Mano et al., 2006). PEX13 interacts with PEX14 and mutation of either protein in *Arabidopsis* results in reduced PTS1 and PTS2 import (Mano et al., 2006; Monroe-Augustus et al., 2011; Woodward et al., 2014). The *Arabidopsis pex13* null allele is lethal, but *pex14* is not (Boisson-Dernier et al., 2008; Monroe-Augustus et al., 2011). In a weak *pex13* mutant, *pex13-4*, the amount of PEX5 associated with the peroxisomal membrane is decreased, but overexpression of *PEX5* can rescue the PTS2-processing defects of *pex13-4* (Woodward et al., 2014), indicating, to some extent, that the role of PEX13 can be bypassed. The *Arabidopsis pex14* mutant shows severe growth defects, but it is still able to complete the life cycle and produce fertile offspring, indicating that PEX14 facilitates, but is not essential for peroxisomal matrix protein import in plants (Monroe-Augustus et al., 2011; Burkhart et al., 2013).

Based on *in vitro* reconstitution and biochemical assays, receptor/cargo complexes are imported into the peroxisome lumen *via* the peroxisomal translocon mainly composed of the receptors and Pex14 in yeasts and mammals (Ma et al., 2009; Meinecke et al., 2010; Romano et al., 2019). Upon arrival in the peroxisome lumen, PTS1 proteins are released from their receptor, Pex5, with the aid of Pex8 in *Hansenula polymorph* (Wang et al., 2003). While in *Pichia pastoris*, the receptor/cargo complex dissociates as a result of a conformational change in Pex5, regulated by redox conditions within the peroxisome lumen and its interaction with Pex8 (Ma et al., 2013). However, neither the constituents of the peroxisomal translocon nor the mechanisms of how cargo is released from receptors have been characterized in plants.

### Dynamic Regulation of the Peroxisomal Importomer

After cargo proteins are released within the peroxisome lumen, the receptors are recycled to the cytosol for another round of import. This process is governed by the monoubiquitination of PEX5 on a cysteine residue, which requires an E1 ubiquitin-activating enzyme, an E2 ubiquitin-conjugating enzyme, and an E3 ligase (Hu et al., 2012; Kao et al., 2018). In yeasts, Pex4, as an E2 ubiquitin-conjugating enzyme, transfers ubiquitin to Pex5 at a conserved cysteine residue (monoubiquitination for recycling) or lysine residue(s) (polyubiquitination for degradation) under the catalysis of Pex2/10/12, three E3 ligases that comprise the RING subcomplex (Platta et al., 2007, 2009).

In *Arabidopsis*, the null mutants of *pex2*, *pex10*, and *pex12* display embryo lethality at the heart stage (Fan et al., 2005; Prestele et al., 2010). Interestingly, the weak mutants of *pex2-1* and *pex10-2* exhibited defects only in the targeting of PTS1 but not PTS2 proteins (Burkhart et al., 2014). Although it has been demonstrated that *Arabidopsis* PEX2, PEX10, and PEX12 contain E3 ubiquitin ligase activity *in vitro*, whether PEX5 is the direct substrate of these E3 ubiquitin ligases has not been directly shown (Kaur et al., 2013). PEX10 has been implicated in PEX5 retrotranslocation because overexpression of PEX5 aggravates the PTS2-processing defects and physiological phenotype of *pex10-2* (Burkhart et al., 2014).

In *Arabidopsis*, the function of PEX4 is enhanced by PEX22, an integral PMP, whose cytosolic domain interacts with PEX4 (El Magraoui et al., 2014). The *Arabidopsis* PEX4-PEX22 complex rescues the growth defect of Pex4/Pex22-deficient yeast cells, indicating that the plant ubiquitin machinery for receptor recycling or degradation is functionally similar to that in the yeast system (Zolman et al., 2005). At normal temperature (22°C), the *pex4* mutant shows elevated levels of membrane-associated PEX5 without ubiquitination, suggesting that ubiquitination is required for PEX5 recycling. But at 28°C, the overall PEX5 protein levels are reduced in the *pex4* mutant, probably resulting from another unknown ubiquitination enzyme promoting the degradation of PEX5 (Kao and Bartel, 2015).

After being monoubiquitinated, PEX5 is exported to the cytosol for another round of import, which requires a membrane-anchored AAA (ATPases associated with various cellular activities) ATPase complex, consisting of PEX1 and PEX6. PEX1 and PEX6 form a heterohexamer with 3 U of each. This complex is anchored to the peroxisomal membrane through a TA protein, Pex26 in yeasts and aberrant peroxisome morphology 9 (APEM9) in *Arabidopsis* (Goto et al., 2011; Gardner et al., 2015). The PEX1-PEX6 complex directly interacts with the ubiquitin moiety of ubiquitinated PEX5 and subsequently releases ubiquitinated PEX5 to the cytosol (Cross et al., 2016; Pedrosa et al., 2018).

In *Arabidopsis*, the RNA interference (RNAi) lines of *pex1i*, *pex6i*, and *apem9i* result in defective PTS1 and PTS2 protein import (Nito et al., 2007; Goto et al., 2011). *pex1-2*, a weak *pex1* mutant, displays severe defects in peroxisome function, while *pex1-3*, a more severe mutant, is embryo lethal, indicating that *Arabidopsis* PEX1 is essential for embryogenesis (Rinaldi et al., 2017). Both *Arabidopsis pex6* and *pex26* mutants display β-oxidation deficiency, impaired matrix protein import, and low levels of PEX5 (Gonzalez et al., 2017). Interestingly, the peroxisomal defects of *pex1*, *pex4*, *pex6*, and *pex26* are aggravated by overexpression of *PEX5* in *Arabidopsis* (Kao and Bartel, 2015; Gonzalez et al., 2017; Rinaldi et al., 2017), indicating that PEX5 functions normally only when it can be recycled efficiently. It has been reported recently that a *pex1* missense mutation, *pex1-1*, can alleviate the physiological defects of *pex6-1* without restoring PEX5 levels, implying additional functions of the PEX1-PEX6 complex beyond PEX5 recycling (Gonzalez et al., 2018). The weak mutant *pex13-1* ameliorates the growth defects of *pex4-1* or *pex6-1* surprisingly, although matrix protein import in *pex4-1 pex13-1* and *pex6-1 pex13-1* remains defective (Ratzel et al., 2011), indicating that normal peroxisomal function is dependent on the balance between import and export of peroxisomal receptors.

In *Arabidopsis*, SP1 (suppressor of plastid protein import locus 1), an E3 ubiquitin ligase located in the peroxisome membrane, is involved in regulating the translocation of PEX5 by participating in the degradation of PEX13 and promoting destabilization of PEX14 and the RING peroxins PEX2, PEX10, and PEX12 (Pan et al., 2016). However, SPL1 (SP1-like 1), a homolog of SP1, stabilizes PEX13 by inhibiting the function of SP1. In contrast to *sp1*, which suppresses the phenotypes of *pex14* and *pex13* mutants, *spl1* enhances the phenotypes of these two mutants (Pan et al., 2018).

In summary, from docking on the peroxisome membrane to being exported to the cytosol, the translocation of *Arabidopsis* PEX5 is dynamically controlled by elaborate mechanisms requiring cooperation among the components of the docking subcomplex, the RING subcomplex, and the receptor recycling machinery. The import of PMPs and matrix proteins are crucial to peroxisome biogenesis. The mechanisms of PMP and matrix protein import are highly conserved but also display specificity among different organisms. The diversity and complexity of the transport system indicates that peroxisomes are highly dynamic and may be differentially regulated during plant development and other various environmental conditions.

## Crosstalk Between Peroxisomes and Other Organelles

Plant peroxisomes are not isolated organelles in cells. They usually communicate and collaborate with other organelles such as lipid bodies, chloroplasts, and mitochondria to execute their multi-faceted functions in biological processes, such as lipid mobilization, photorespiration, and redox metabolism (Shai et al., 2016).

In plants, lipid bodies accumulate in seeds and cotyledons that generally store lipids as triacylglycerols (TAGs). During seed germination, fatty acids are transported from lipid bodies to glyoxysomes and subsequently metabolized through fatty acid β-oxidation and the glyoxylate cycle to supply energy for seedling establishment (Graham, 2008; Hu et al., 2012). The transport of fatty acids may be mediated by the direct contact between glyoxysomes and oil body. In *Arabidopsis*, the *ped1* mutant, which has a defect in a thiolase gene involved in fatty acid β-oxidation, grows slowly in the dark with small cotyledons and displays abnormal glyoxysomes with tubular structures which are derived from the invagination of glyoxysomes membrane (Hayashi et al., 2001). Fatty acid-containing vesicles in the tubular compartments are formed at the contact sites of lipid bodies and glyoxysomes, suggesting that there is a direct mechanism of lipid transport from the lipid bodies to glyoxysomes (Hayashi et al., 2001).

The enzymes involved in TAG degradation can also be transferred between the peroxisome and oil body. During seed germination, TAGs are hydrolyzed by lipases to free fatty acids and glycerol, which results in the degradation of lipid bodies. During oil mobilization, *Arabidopsis* SDP1, a major TAG lipase, which is first localized to the peroxisome membrane, migrates to the surface of lipid bodies for oil mobilization *via* extensive tubulations of the peroxisome known as peroxules (Thazar-Poulot et al., 2015). The movement of SDP1 from the peroxisome to lipid bodies requires a retromer complex, which is a protein complex mediating the recycling of receptors and the retrograde transport of cargo proteins from endosomes to the trans-Golgi network (Bonifacino and Hurley, 2008). In core retromer mutants, oil body biogenesis and oil breakdown are defective, suggesting that the contact between glyoxysomes and oil body is important for oil mobilization (Thazar-Poulot et al., 2015).

In addition to oil bodies, peroxisomes are closely related to other organelles. For example, EM images showed that plant peroxisomes are localized close to chloroplasts and mitochondria (Kaur et al., 2009). The close contact between peroxisome and these two organelles plays an important role in the metabolism of plant cells. In prolonged dark conditions, free fatty acids are released from the chloroplast and subsequently metabolized *via* peroxisomal β-oxidation. During this process, PXA1 mediates the import of fatty acids into peroxisomes and KAT2 (PED1) is involved in β-oxidation (Germain et al., 2001; Zolman et al., 2001; Kunz et al., 2009; Hu et al., 2012).

Plant photorespiration requires the coordination of four compartments: the cytosol, chloroplasts, mitochondria, and peroxisomes. Conjunction of peroxisomes with chloroplasts and mitochondria may play an important role in photorespiration by allowing for metabolite flow between these organelles (Hu et al., 2012). Recent evidence obtained from light and transmission electron microscopy show that peroxisomes are appressed to the chloroplast envelope in *Arabidopsis.* The RING finger domain in PEX10 is crucial for the contact between these two organelles. Expressing endogenous levels of *PEX10* with a dysfunctional RING finger domain results in more numerous, multilobed, clustered peroxisomes, which are not associated with the chloroplast envelope as they are in wild type (Schumann et al., 2007). Moreover, peroxisomes can alter their shape from spherical to elliptical to increase the interaction area with the chloroplast upon exposure of the plant to light (Oikawa et al., 2015).

Peroxisomes, chloroplast, and mitochondria not only cooperate in metabolic processes but also in division (Zhang and Hu, 2010; Aung and Hu, 2011). Peroxisome proliferation first requires the elongation of a preexisting peroxisome, followed by constriction and fission (Schrader, 2006). In *Arabidopsis*, the PEX11 family, consisting of five partially functionally redundant isoforms Pex11a to Pex11e (Orth et al., 2007; Desai and Hu, 2008), is involved in peroxisome elongation (Lingard and Trelease, 2006; Koch et al., 2010). Subsequently, peroxisome fission is dependent on the coordination of integral membrane-anchored proteins FIS1 (FISSION1) and dynamin-related protein (DRP). The two FIS1 homologs, FIS1A and FIS1B, are targeted to both peroxisomes and mitochondria to facilitate the division of both organelles (Scott et al., 2006; Zhang and Hu, 2008). FIS1 interacts with DRP3 to associate with the both peroxisome and mitochondria membranes. DRP3A and DRP3B, which are the constituents of DRP3, are partially redundant in mediating the division of peroxisomes and mitochondria (Mano et al., 2004; Zhang and Hu, 2008; Fujimoto et al., 2009). DRP5B is involved in the division of peroxisomes, chloroplasts, and mitochondria, which is independent of DRP3 (Aung and Hu, 2012). Another factor, a coiled-coil protein peroxisomal and mitochondrial division factor 1 (PMD1), whose function is independent of FIS1 and DRP3, is also tethered to the membranes of peroxisomes and mitochondria to regulate the division of the two organelles (Aung and Hu, 2011).

## Peroxisomes Function as Sensors of Environmental Redox Changes

Peroxisomes are important for modulating redox balance between ROS production and elimination. In plants, stress conditions promote a ROS-dependent signaling pathway which triggers rapid and adaptive responses (Sandalio and Romero-Puertas, 2015). The excess oxidative stress induced in these conditions can affect the biogenesis and degradation of peroxisomes (Shibata et al. 2013; Wang et al., 2015).

### Peroxisome Status Is Affected by Environmental Stresses

Because of their immobility, plants are constantly suffering from stresses caused by environmental changes. Abiotic and biotic stresses, such as drought, high and low temperatures, salinity, nutrient deficiency, and pathogens are the main factors leading to crop yield loss (Zhang et al., 2010, 2018; Ren et al., 2013; Shen et al., 2014). In general, environmental stresses induce cellular oxidative stress and as a consequence induce the ROS scavenging system (Qi et al., 2010; Li et al., 2012; Liu et al., 2017). Peroxisomes are crucial in this ROS scavenging system for controlling H_2_O_2_ levels (Mhamdi et al., 2012).

Peroxisomes are one of the main sites of H_2_O_2_ generation in plant cells. Plants peroxisomes also contain several enzymes which eliminate excess H_2_O_2_, including catalases, ascorbate peroxidases (APX), and various types of peroxiredoxins (Schrader and Fahimi, 2006; Mhamdi et al., 2010). Among these enzymes, catalases are the main eliminator of peroxisomal H_2_O_2_. In *Arabidopsis*, there are three catalase genes (*CAT1*, *CAT2*, and *CAT3*) which are involved in many plant physiological processes including abiotic stresses response (Su et al., 2018).

In regard to abiotic stresses, *Arabidopsis* salt overly sensitive 2 (SOS2), which is required for salt tolerance, can interact with CAT2 and CAT3 at an unknown subcellular location in response to salt stress and H_2_O_2_ metabolism (Verslues et al., 2007). A zinc finger protein lesion simulating disease 1 (LSD1) can interact with all three catalases in the cytosol to maintain their activities in regulating hypersensitive cell death (Li et al., 2013). Moreover, the chaperone no catalase activity 1 (NCA1), which interacts with all three catalases in the cytosol, is required for catalase activity during multiple stress responses, such as salt, cold, and alkaline stress, as well as for autophagy-dependent cell death (Hackenberg et al., 2013; Li et al., 2015). In addition, a peroxisome-localized small heat shock protein Hsp17.6CII interacts with CAT2 in the peroxisome and enhances CAT2 activity to protect from abiotic stresses (Li et al., 2017).

For biotic stresses, it is reported that *Arabidopsis* PEN2, a glycosyl hydrolase localized to both peroxisomes and mitochondria, acts as a component of an inducible preinvasion resistance system to restrict pathogen entry *via* the pathogen-induced enzymatic activation of indole glucosinolates (Lipka et al., 2005; Fuchs et al., 2016). Glucosinolates are secondary metabolites and are often stored in the vacuole as inactive glycoside precursors. These precursors can be activated by PEN2 when plants undergo insect feeding or necrotrophic pathogen attack (Bednarek et al., 2009). In addition, viruses can produce viral suppressors of RNA silencing (VSR) to protect themselves against RNAi, an important plant defense mechanism mediated by small interfering RNA. The peanut clump virus-encoded P15, which is one such VSR, transports the antiviral siRNA from the cytosol to the peroxisomal matrix to potentiate viral infection (Incarbone et al., 2017).

Different stresses often affect the expression of peroxisome biogenesis genes. In *Arabidopsis*, *PEX1*, *PEX5*, *PEX10*, and *PEX14* are significantly induced by exogenous H_2_O_2_. In addition, pathogen attack and wounding, which trigger an oxidative burst, rapidly induce *PEX1* expression, indicating that peroxisome biogenesis is directly responsive to stresses (Lopez-Huertas et al., 2000).

Various environmental conditions, which are often accompanied by ROS accumulation, can also trigger peroxisome proliferation to respond to the changes. During seedling photomorphogenesis, light induces peroxisome proliferation by upregulating *PEX11b* through the HYH (HY5 HOMOLOG)-mediated light signaling cascade. In this process, the activity of PEX11b depends on the far-red light receptor phytochrome A and the bZIP transcription factor HYH (Desai and Hu, 2008). In addition to directly targeting the *PEX11b* promoter to induce its expression, HYH can also indirectly repress the activity of FHA3 (forkhead-associated domain protein 3), which negatively regulates *PEX11b* expression and peroxisome division (Desai and Hu, 2008; Desai et al., 2017). In *Pisum sativum L.* plants, peroxisome proliferation can be induced by clofibrate, which increases activated oxygen species and lipid peroxidation of peroxisomal membranes (Palma et al., 1991). In addition, peroxisome proliferation can also be induced under clofibrate treatment in *Arabidopsis* (Castillo et al., 2008). Cadmium-imposed oxidative stress can also induce an increase in the number of peroxisomes and induces formation of peroxisome peroxules regulated by *PEX11a* (Rodríguez-Serrano et al., 2009, 2016). Peroxules can also be induced under high light stress accompanied by accumulation of H_2_O_2_ (Jaipargas et al., 2016). In addition, the number of peroxisomes in *Arabidopsis* is increased when exposed to high salt (Fahy et al., 2017). Under salt stress, mitogen-activated protein kinase 17 (MPK17) negatively regulates peroxisome proliferation by inhibiting PMD1 (Frick and Strader, 2018). Together, in addition to the role of ROS in signaling, these evidences suggest that ROS induced by primary environmental stress are also involved in peroxisome proliferation by regulating the expression of *PEX* genes.

### Redox State Regulates Pexophagy and Peroxisome Remodeling

The number and activity of peroxisomes are tightly controlled and maintained in a balanced state in response to different environmental and/or physiological conditions. Peroxisome homeostasis is achieved through the coordination of peroxisome biogenesis and turnover. Pexophagy, a selective autophagy pathway to remove peroxisomes, is critical for peroxisomal quality control and requires a suite of *autophagy-related* (*ATG*) genes. These ATGs are highly conserved in yeasts, animal, and plants (Li and Vierstra, 2012).

Under oxidative stress, pexophagy is enhanced to remove oxidative, damaged peroxisomes. In *Arabidopsis*, the peroxisomes in *atg2*, *atg7*, and *atg18* mutants aggregate together with accumulation of inactive catalases in the clustered peroxisomes, indicating these peroxisomes are ROS-damaged organelles to be degraded (Shibata et al., 2013). Aggregated peroxisomes are also observed in the *cat2* mutant (Shibata et al., 2013). ATG8, the autophagosome marker, colocalizes with the aggregated peroxisomes, indicating that autophagy is involved in the selective degradation of damaged peroxisomes (Shibata et al., 2013). Pexophagy rates differ depending on the plant tissue. Increased peroxisome abundance in *Arabidopsis atg2*, *atg5*, *atg7*, and *atg9* mutants are detected in hypocotyls and leaves but not roots (Yoshimoto et al., 2014). This phenomenon may be because the above ground part of plants is the main place for H_2_O_2_ production, resulting in more oxidatively damaged peroxisomes to be degraded by pexophagy (Yoshimoto et al., 2014).

Pexophagy also plays a crucial role in peroxisome remodeling. In plant seedlings, the peroxisomes, named glyoxysomes, contain enzymes such as thiolase involved in β-oxidation and two main glyoxylate cycle enzymes, isocitrate lyase (ICL) and malate synthase (MLS), which catalyze the conversion of fatty acids into carbohydrates (Pracharoenwattana and Smith, 2008). As the seedlings grow and photosynthesis is established, the enzymes involved in the glyoxylate cycle become unnecessary and are degraded. At the same time, redundant or obsolete glyoxysomes are degraded by pexophagy while some glyoxysomes are transformed into leaf peroxisomes harboring photorespiration enzymes (Goto-Yamada et al. 2014). In *Arabidopsis atg5* and *atg7* mutants, the degradation of ICL and MLS are delayed during growth in hypocotyls but not in whole seedlings, indicating pexophagy may promote degradation of peroxisomes and contribute to the degradation of peroxisome matrix proteins (Kim et al., 2013).

The degradation of ICL and MLS by pexophagy was nicely elucidated by screening for suppressors of the *lon2* mutant. Lack of LON2 causes inefficient β-oxidation of indole-3-butyric acid (IBA) into indole-3-acetic acid (IAA), defective in PTS2 proteins targeting, such as thiolase and peroxisomal malate dehydrogenase (PMDH), reduced matrix protein import, and clustered peroxisomes, indicating that LON2 plays crucial roles in sustaining normal peroxisomal function (Lingard and Bartel, 2009; Farmer et al., 2013; Shibata et al., 2013; Young and Bartel, 2016). In the *lon2* mutant, ICL and MLS are unstable. Mutation of autophagy genes such as *ATG2*, *ATG3*, and *ATG7* in *lon2* mutants result in stabilization of ICL and MLS, suggesting that pexophagy participates in degradation of peroxisomal proteins during peroxisome remodeling and is induced when LON2 is nonfunctional (Farmer et al., 2013; Goto-Yamada et al., 2014).

Damaged or obsolete ICL and MLS proteins can also be degraded through peroxisome-associated protein degradation (PexAD) pathway (Lingard et al., 2009). In the peroxisome matrix protein import mutants *pex5* and *pex14*, ICL and MLS are stabilized, suggesting ICL and MLS must be localized in peroxisomes to be degraded (Lingard et al., 2009; Burkhart et al., 2013). Moreover, the turnover of ICL and MLS is affected by peroxisomal metabolism. In *pxa1* and *ped1* mutants, ICL and MLS are stabilized, potentially as a result of decreased H_2_O_2_ levels due to blocked fatty acid entry into peroxisomes and reduced β-oxidation (Kunz et al., 2009; Lingard et al. 2009; Burkhart et al., 2013). Interestingly, the degradation of ICL and MLS is enhanced in the *cat2* mutant, where peroxisomes are more oxidative due to the lack of a H_2_O_2_-scavenging catalase (Lingard et al., 2009). These results indicate that redox status modulates the degradation rate of ICL and MLS. Furthermore, ICL and MLS are more stabilized in *pex4*, *pex22*, and *pex6* mutants, implying that the ubiquitin-proteasome pathway is required for the degradation of ICL and MLS and implicated in peroxisome remodeling (Lingard et al., 2009; Burkhart et al., 2013).

In addition to the degradation of ICL and MLS, the conversion of glyoxysomes to leaf peroxisomes also requires the uptake of photorespiratory enzymes such as hydroxypyruvate reductases (HPR) (Lingard et al., 2009). However, the level of HPR in *lon2*, *atg7*, and *lon2 atg7* mutants is similar to that in wild-type *Arabidopsis*, suggesting that HPR synthesis is independent of ICL and MLS abundance and the import of HPR occurs after the formation of leaf peroxisomes (Farmer et al., 2013).

The receptors for pexophagy are Atg30 (*Pichia pastoris*)/Atg36 (*S. cerevisiae*) in yeasts and NBR1 in mammals (Farre et al., 2008; Motley et al., 2012). A plant homolog of NBR1 has been identified, but there is no direct evidence to show it is a pexophagy receptor (Svenning et al., 2011; Young et al., 2019).

### Redox State Regulates Retrograde Signaling From Peroxisomes to the Nucleus

**Figure 2:**
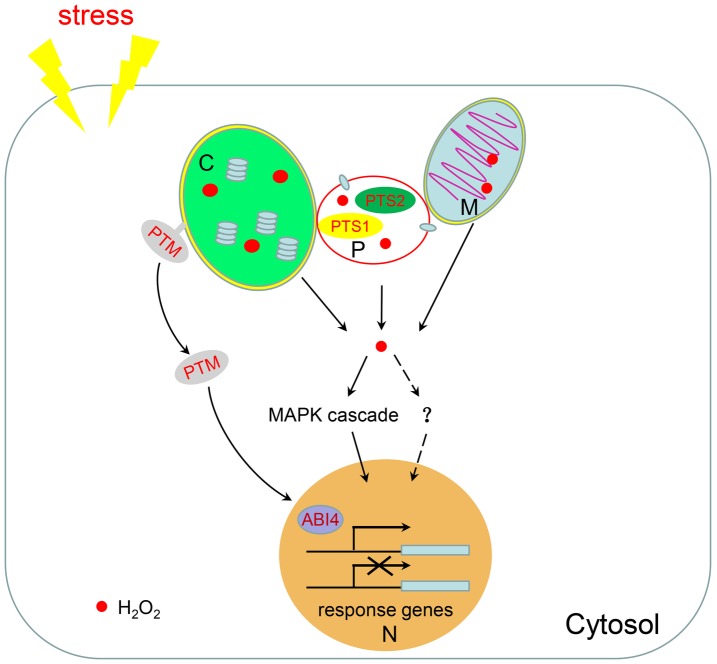
H_2_O_2_-mediated retrograde signaling. Chloroplasts, mitochondria, and peroxisomes are linked together to facilitate the shuttling of intermediate metabolites between them. When plants are subjected to environmental stresses, a large amount of H_2_O_2_ is produced in these three organelles. H_2_O_2_ can be used as a signal molecule to regulate the expression of nuclear response genes through the MAPK cascade or potentially through other unknown pathways. H_2_O_2_ in chloroplasts also promotes the dissociation of the transcription factor PTM from the chloroplast membrane to regulate nuclear gene expression. M, mitochondria; C, chloroplast; P, peroxisome; N, nucleus.

Intracellular signaling from organelles to the nucleus, termed retrograde signaling, can modulate the expression of nuclear genes, which play important roles in organelle assembly, metabolism, and response to environmental changes (Sewelam et al., 2014). In plants, ROS are produced in chloroplasts, mitochondria, cytosol, peroxisomes, and the plasma membrane (Das and Roychoudhury, 2014; de Souza et al., 2017). The cellular movement and diffusion of H_2_O_2_ is proposed to be facilitated by aquaporins (Bienert and Chaumont, 2014). It has been hypothesized that H_2_O_2_ signaling could result in two kinds of responses: (1) the signaling induced by H_2_O_2_ is integrated regardless of the origin of H_2_O_2_ or (2) H_2_O_2_-induced signaling is dependent on the production site of H_2_O_2_ (Sewelam et al., 2014). Given that the production of ROS occurs in multiple organelles, it is necessary to discuss the different effects of ROS signals derived from different organelles (Figure 2).

The peroxisome-derived signaling pathway has emerged by analyzing the catalase mutants which cause accumulation of H_2_O_2_ in peroxisomes. In tobacco and *Arabidopsis*, catalase-deficient lines display enhanced photorespiratory H_2_O_2_ levels, which trigger induction of pathogenesis-related (PR) genes and cell death (Takahashi et al., 1997; Chaouch et al., 2010). Under high light conditions, catalase-deficient plants provoke significant differences in nuclear gene expression profiles (Vandenabeele et al., 2003, 2004). In addition, large-scale transcriptomic analysis of the *cat2* mutant demonstrated that H_2_O_2_ produced from peroxisomes induces prevailing protein repair responses (Queval et al., 2007). A more profound transcriptional change is observed in the *cat1 cat2 cat3* triple mutant with the differentially expressed genes (DEGs) being enriched in those encoding transcription factors and receptor-like protein kinases (Su et al., 2018). In addition, expression of *oxidative signal inducible 1* (*OXI1*), a gene encoding serine/threonine kinase, and several genes of the MAPK cascade pathway, such as *MPK11* and *MPK13*, are dramatically changed in the *cat1 cat2 cat3* triple mutant (Su et al., 2018). It has been reported that the OXI1-dependent pathway involving MPK3/6 can be activated by exogenous H_2_O_2_ (Rentel et al., 2004). Thus, it seems that signaling pathways activated by different sources of H_2_O_2_ may converge on OXI1, but the downstream signaling pathways are context-dependent.

The ROS-induced chloroplast retrograde signaling pathway is mediated by PTM, a plant homeodomain-type transcription factor with transmembrane domains which localizes in the chloroplast envelope (Sun et al., 2011). Under high light, proteolytic cleavage of PTM enables the release of the PTM N-terminus from the chloroplast envelope, which is then transferred and accumulated in the nucleus to regulate the expression of photosynthesis-associated nuclear genes including *ABI4* (abscisic acid insensitive 4). In turn, this causes repression of the expression of *LHCB*, which encodes the photosynthesis-related light-harvesting complex II chlorophyll-a/b binding protein that is involved in photosynthetic generation of reducing power by harvesting solar energy (Sun et al., 2011). In *Nicotiana benthamiana*, high light induces chloroplast-derived H_2_O_2_ to be transferred to the nucleus, where they affect the nuclear redox state and gene transcription (Exposito-Rodriguez et al., 2017). During plant innate immunity responses, stromules, tubular extensions from the chloroplast, are induced and surround the nucleus to connect with it. Stromule formation is correlated with increased SA and H_2_O_2_ (Caplan et al., 2015). Chloroplast-sourced H_2_O_2_ and the chloroplast-localized defense protein NRIP1 (N receptor interacting protein 1) can move to the nucleus *via* stromule connections, indicating that stromules participate in chloroplast-to-nuclear retrograde signaling (Caplan et al., 2008, 2015).

As in chloroplasts, ROS is also involved in mitochondrial retrograde-signaling cascades. As a key component of chloroplast retrograde signaling pathway, ABI4 also plays a central role in mediating mitochondrial retrograde signaling by inducing *alternative oxidase 1a* (*AOX1a*). *AOX1a* encodes a mitochondrial inner membrane protein acting as a terminal electron acceptor of the alternative pathway in the electron transport chain. It is an important sensor of oxidation levels in cells and plays significant roles in maintaining the cellular redox state by regulating the malate valve and the antioxidative systems and sustaining photosynthesis by regulating the redox state of chloroplastic electron transport mediators and non-photochemical quenching (NPQ) in *Arabidopsis* (Giraud et al., 2009; Vishwakarma et al., 2014). In *Arabidopsis*, regulator of alternative oxidase 1 (RAO1) has been identified as the nucleus-localized cyclin-dependent kinase E1 that regulates the expression of both *LHCB* and *AOX1a* to integrate chloroplast and mitochondrial retrograde signals (Ng et al., 2013; Blanco et al., 2014). These results illustrate the coordination of these two organelles in retrograde signal pathways.

It has been reported that H_2_O_2_ generated from chloroplasts and peroxisomes induce different transcriptome profiles (Sewelam et al., 2014). H_2_O_2_ produced from chloroplasts in *Arabidopsis* glycolate oxidase (GO) overexpressing lines induces expression of genes involved in signaling responses, such as genes encoding transcription factors, protein/receptor kinases, and defense proteins, and genes involved in biosynthesis of secondary signaling messengers (Sewelam et al., 2014). By contrast, H_2_O_2_ produced from peroxisomes in *Arabidopsis* catalase-deficient mutants induces a stress recovery response by inducing genes encoding heat shock proteins (HSPs) and proteins involved in ubiquitin-dependent protein degradation (Sewelam et al., 2014). However, a group of genes are induced by H_2_O_2_ produced in both the chloroplast and peroxisome, indicating that the response induced by H_2_O_2_ can depend on either the origination of the H_2_O_2_ in specific organelles or on downstream integration of the H_2_O_2_ signals independent from the site of production (Sewelam et al., 2014). There are more evidence supports this view. According to microarray-based transcriptomic analysis, ^1^O_2_ derived in the chloroplasts of the chlorophyll b-deficient mutant *chlorina1* (*ch1*) results in 302 genes expressed differentially compared to wild-type, far fewer than the number of DEGs (2,852 genes) observed in the *cat1/2/3* mutant compared to wild-type. Interestingly, 43.7% of the DEGs in *ch1* are shared with those found in the catalase triple mutant, when compared to wild type (Ramel et al., 2013; Su et al., 2018). The ROS signals derived from chloroplasts, mitochondria, and peroxisomes are proposed to connect in the cytoplasm and link to the MAPK pathway to regulate the expression of nuclear genes (Noctor and Foyer, 2016; Shumbe et al., 2016). However, to what extent these pathways connect to each other and the detailed molecular mechanisms remain unclear.

## Prospects

Peroxisome homeostasis is regulated by complex regulatory systems. Recent progress has been made in understanding peroxisome biogenesis and degradation and how peroxisomes communicate with other organelles; however, many questions are still unresolved. For example, does *de novo* peroxisome formation exists in plants? What is the physiological importance of peroxisomes being connected to other organelles? What components comprise the membrane contact sites? What are the molecular mechanisms underlying peroxisome-derived retrograde signaling? Is ROS-mediated organellar retrograde signaling pathway integrated or organelle specific? Lastly, the pexophagy receptor in plants has yet to be identified. We hope these problems will be solved in the next few decades.

## Author Contributions

TS and CM conceived and wrote the manuscript. WL and PW contributed to the revision of the manuscript. All authors read and approved the submitted version.

### Conflict of Interest Statement

The authors declare that the research was conducted in the absence of any commercial or financial relationships that could be construed as a potential conflict of interest.
